# Impact of race-independent equations on estimating glomerular filtration rate for the assessment of kidney dysfunction in liver disease

**DOI:** 10.1186/s12882-023-03136-y

**Published:** 2023-03-31

**Authors:** Frank Stämmler, Laurence Derain-Dubourg, Sandrine Lemoine, Jeffrey W. Meeusen, Surendra Dasari, John C. Lieske, Andrew Robertson, Eric Schiffer

**Affiliations:** 1 Department of Research and Development, Numares AG,, Am BioPark 9, 93053 Regensburg, Germany; 2 Department Néphrologie, Dialyse, Hypertension Et Exploration Fonctionnelle Rénale, Groupement Hospitalier Edouard Herriot, Hospices Civils de Lyon, Université Claude Bernard, Lyon 1, Lyon, France; 3 Department of Laboratory Medicine and Pathology, Mayo Clinic, Rochester, MN USA; 4 Division of Nephrology and Hypertension, Mayo Clinic, Rochester, MN USA

**Keywords:** eGFR, GFR_NMR_, Creatinine, Cystatin C, Chronic liver disease

## Abstract

**Background:**

Altered hemodynamics in liver disease often results in overestimation of glomerular filtration rate (GFR) by creatinine-based GFR estimating (eGFR) equations. Recently, we have validated a novel eGFR equation based on serum myo-inositol, valine, and creatinine quantified by nuclear magnetic resonance spectroscopy in combination with cystatin C, age and sex (GFR_NMR_). We hypothesized that GFR_NMR_ could improve chronic kidney disease (CKD) classification in the setting of liver disease.

**Results:**

We conducted a retrospective multicenter study in 205 patients with chronic liver disease (CLD), comparing the performance of GFR_NMR_ to that of validated CKD-EPI eGFR equations, including eGFRcr (based on creatinine) and eGFRcr-cys (based on both creatinine and cystatin C), using measured GFR as reference standard. GFR_NMR_ outperformed all other equations with a low overall median bias (-1 vs. -6 to 4 ml/min/1.73 m^2^ for the other equations; *p* < 0.05) and the lowest difference in bias between reduced and preserved liver function (-3 vs. -16 to -8 ml/min/1.73 m^2^ for other equations). Concordant classification by CKD stage was highest for GFR_NMR_ (59% vs. 48% to 53%) and less biased in estimating CKD severity compared to the other equations. GFR_NMR_ P30 accuracy (83%) was higher than that of eGFRcr (75%; *p* = 0.019) and comparable to that of eGFRcr-cys (86%; *p* = 0.578).

**Conclusions:**

Addition of myo-inositol and valine to creatinine and cystatin C in GFR_NMR_ further improved GFR estimation in CLD patients and accurately stratified liver disease patients into CKD stages.

**Supplementary Information:**

The online version contains supplementary material available at 10.1186/s12882-023-03136-y.

## Introduction

Chronic liver disease (CLD) is commonly associated with impaired kidney function. Renal dysfunction in the context of CLD is a predictor of mortality [1], and also a strong prognostic predictor of orthoptic liver transplantation (OLT) outcomes [2]. The influence of renal dysfunction is so well defined that it is a component of Model of End stage Liver Disease (MELD) prognostic score. Furthermore, the degree of renal dysfunction in CLD has wide-reaching clinical decision implications such as appropriate drug dosing, therapeutic interventions, and suitability for OLT [3, 4]. Most treatments for complications of liver disease, such as nephrotoxic antibiotics, diuretics, and paracentesis, have further negative effects on renal function with the potential to precipitate or aggravate renal failure [5]. An accurate measure of renal function is therefore crucial in patients with CLD.

Tracer-measured glomerular filtration rate (mGFR) is considered the gold standard for determining GFR. However, it is not readily available in most centers. Hence, renal function is estimated using biomarker-based eGFR equations in clinical routine settings. The most current eGFR equations are based on models containing either serum creatinine (cr), cystatin C (cys), or both. The leading examples are the Chronic Kidney Disease Epidemiology Collaboration (CKD-EPI) eGFR equations, which employ creatinine, age (A), sex (S), with or without race (R) [eGFRcr(ASR) or eGFRcr(AS)] or both creatinine and cystatin C [eGFRcr-cys(ASR) or eGFRcr-cys(AS)] [6–8]. Unfortunately, serum creatinine is inaccurate in the diagnosis of renal dysfunction in patients with CLD. Clinical features of this patient population, such as a reduction of total muscle mass, a reduced hepatic conversion of creatine to creatinine due to liver insufficiency, altered hemodynamics in ascites, and an increased tubular secretion rate of creatinine are all likely to account for the failure of serum creatinine levels to increase despite obvious renal disease [9–11]. Additionally, numerous studies have demonstrated the poor performance of creatinine-based eGFR equations in patients with CLD and those being considered for OLT [5, 12, 13].

The alternative biomarker cystatin C, being a non-glycosylated basic protein produced at a constant rate by all nucleated cells, has been introduced to estimate GFR [7]. Unlike creatinine, cystatin C is less influenced by body mass, sex, age, or serum bilirubin, and is mainly influenced by GFR. Therefore, cystatin C is considered to be a superior GFR marker that can provide a more accurate prediction of GFR than creatinine [14]. Also, studies have demonstrated the superiority of estimating GFR using cystatin C in CLD patients compared to creatinine, and particularly the eGFRcys(ASR) for patients with ascites or significant renal disease [15]. Moreover, a recent meta-analysis in cirrhosis patients indicated that eGFR equations based on both creatinine and cystatin C were less biased than those based on creatinine alone, which overestimate GFR, and cystatin C alone, which tend to underestimate GFR [16].

Recently, an eGFR equation has been developed and validated based on nuclear magnetic resonance (NMR) measurement of serum myo-inositol, valine, and creatinine, in addition to the immunoturbidometric quantification of serum cystatin C, age and sex (GFR_NMR_) [17, 18]. NMR represents a multiplex analyzer capable to precisely quantify multiple unlabeled metabolites in a simultaneous physical measurement step. In that sense, GFR_NMR_ interprets biomarkers of glomerular filtration rate in combination with biomarkers reflecting CKD associated metabolic comorbidities. In addition, the quantification of serum creatinine in adjunct to serum valine and myo-inositol by NMR limits the complexity of working streams and associated analytical costs in routine laboratory settings compared to multiple single biomarker assays. Upon clinical validation in adults with and without chronic kidney disease (CKD), GFR_NMR_ showed a lower median bias to tracer mGFR and a higher accuracy within 15% of mGFR, compared to the eGFRcr(ASR), eGFRcys(ASR), and eGFRcr-cys(ASR) CKD-EPI equations [17].

Given this noted superior performance of GFR_NMR_ in adults with and without CKD, this multicenter retrospective study sought to compare the performance of GFR_NMR_ with that of CKD-EPI equations based on creatinine only [eGFRcr(ASR), eGFRcr(AS)] or on both creatinine and cystatin C [eGFRcr-cys(ASR), eGFRcr-cys(AS)], against mGFR as reference standard, in 205 patients with CLD.

## Patients and methods

### Patients and samples

Bio-banked serum samples of adult individuals ≥ 18 years old with CLD were included in this study. These samples were a subset of those described by Stämmler et al. [17], and were selected from patients with CLD within the validation cohort (i.e., not part of the development dataset) [17]. A total of 205 samples were included in this analysis, collected from CLD patients in Rochester, MN, USA (*n* = 155) and Lyon, France (*n* = 50). Samples were stored at − 80 °C and underwent no more than one freeze–thaw cycle before NMR analysis, as previously described [17]. The study was conducted according to the guidelines of the Declarations of Helsinki and Istanbul, and was approved by the relevant Institutional Review Boards (Mayo Clinic IRB# 19–003,513, dated 16 May 2019, and Hospital Edouard Herriot IRB# DC-2012–1615, dated 2 July 2012. All individuals gave informed consent before joining the study.

### mGFR, biomarker measurements and eGFR

Measured GFR (mGFR) reference standard was determined using iothalamate (Rochester samples) or inulin (Lyon samples) clearance, as previously described [15, 17]. Serum creatinine was measured using enzymatic methods traceable to the National Institute of Standards and Technology, as described [17]. Serum cystatin C was measured using immunoassays, as explained [17]. NMR-based measurement and quantification of serum creatinine, myo-inositol and valine were conducted as reported [17–19]. For this work we compared the performance of our recently introduced GFR_NMR_ equation [17, 18] to that of the different CKD-EPI eGFR equations: the 2009 creatinine-based CKD-EPI equation [eGFRcr(ASR); [6]], the 2012 creatinine- and cystatin C-based CKD-EPI equation [eGFRcr-cys(ASR); [7]], and the 2021 race-free derivatives of the previous CKD-EPI equations [eGFRcr(AS) and eGFRcr-cys(AS); [8]].

### Liver function scoring and definitions

**Fig. 1:**
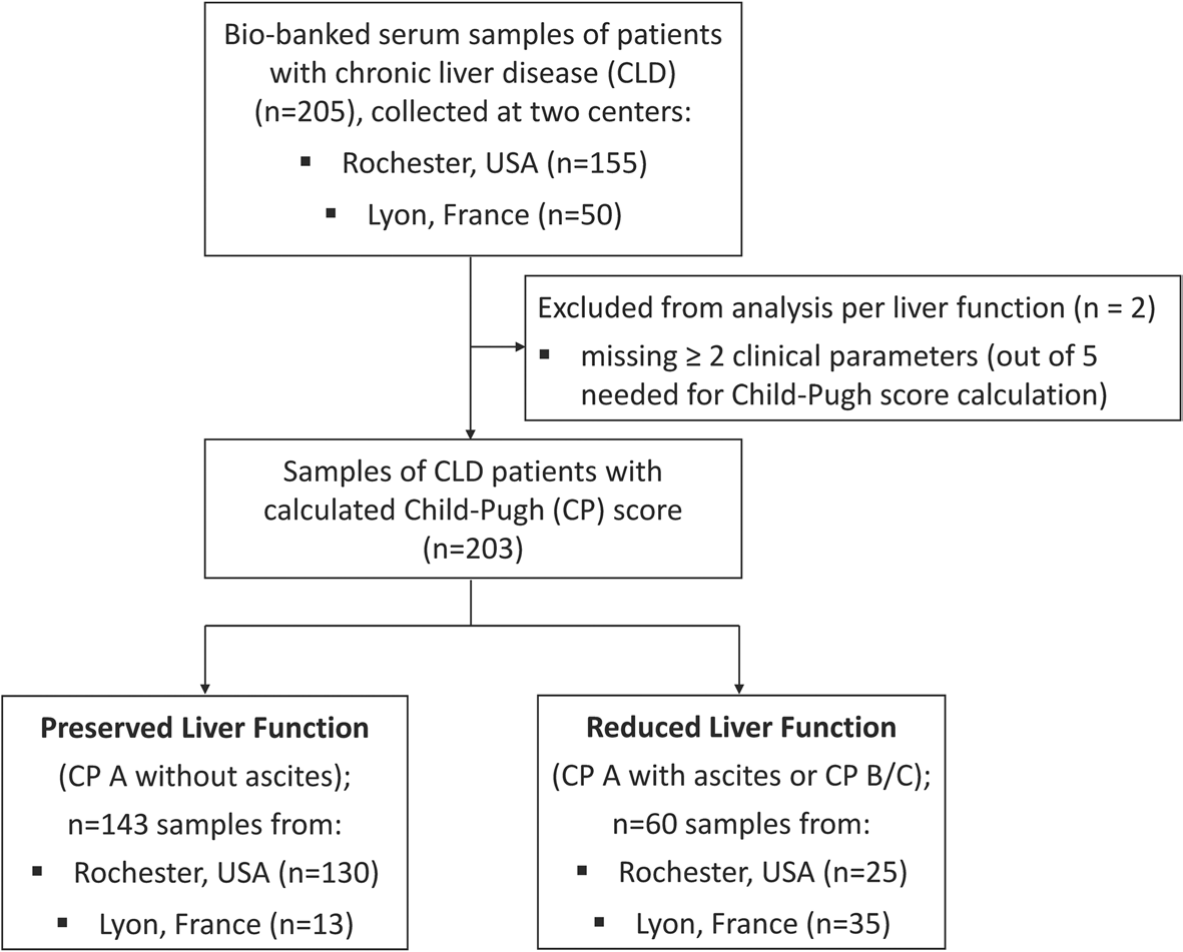
Study flow diagram. Global performance analyses were conducted on all samples of patients with chronic liver disease (CLD; *n* = 205), while analyses according to liver function were conducted in CLD patients with a calculated Child–Pugh (CP) score (*n* = 203). Out of 203 samples with a CP score, 143 were defined a ‘preserved liver function’ (CP class A without ascites) and 60 were defined a ‘reduced liver function’ (CP class A with ascites, CP class B, or CP class C). The number of samples from the respective centers (Lyon, Rochester) is indicated

The performance of eGFR equations was evaluated according to liver dysfunction severity. Two levels of liver function, namely ‘preserved liver function’ and ‘reduced liver function’, were defined based on the Child–Pugh (CP) score [20, 21] and the presence or absence of ascites. Preserved liver function was defined by a CP class A without ascites. Reduced liver function was defined by a CP class A with ascites, or a CP class B or C (regardless of the presence of ascites) (Fig. 1).

CP scores of the Rochester cohort were retrospectively calculated based on available clinical parameters [20, 21]. CP scoring was harmonized across the cohorts. Ascites was considered as either absent or present (‘mild/moderate’ and ‘severe/permanent’ pooled categories). One score point was attributed in the absence of ascites and two points in its presence. In addition, presence, but not severity, of hepatic encephalopathy (HE) was documented at both centers. Therefore, one score point was assigned to samples without HE and two points for samples with HE. In case one out of the five clinical parameters required for CP score calculation was missing, one score point was arbitrarily attributed to the missing parameter to allow calculation of a CP score. In case more than one clinical parameter was missing, samples were excluded from analyses based on liver function, but included in the global performance analyses (Fig. 1).

### Other variables

The performance of eGFR equations was also evaluated in terms of correct CKD staging (G1 to G5) [22], using mGFR as reference, in the whole population, in preserved versus reduced liver function subgroups, and according to the following liver and renal dysfunction parameters: (i) the severity of liver dysfunction based on the Model of End-stage Liver Disease (MELD) score [21, 23], with a threshold of MELD > 15 for liver dysfunction, (ii) the presence or absence of ascites, and (iii) renal dysfunction (mGFR < 60 ml/min/1.73 m^2^). Other relevant clinical parameters are listed in Table 1.

**Table 1:** Patients’ characteristics according to liver function. (To be placed after ‘Results’ subsection ‘Patient’s characteristics’)

**Characteristics**	**N**^a^	**Preserved Liver Function,**^b^** N = 143**	**Reduced Liver Function,**^c^**N = 60**	***p*-value**^d^	**q-value**^e^
Center, n / N (%)	203			< 0.001	** < 0.001**
Lyon (*n* = 48)		13 / 143 (9.0%)	35 / 60 (58.0%)		
Rochester (*n* = 155)		130 / 143 (91.0%)	25 / 60 (42.0%)		
Age, Mean (SD) (years)	203	58.5 (12.6)	58.0 (10.0)	0.3	0.4
Age Group, n / N (%)	203			0.029	**0.049**
< 40 years		12 / 143 (8.4%)	4 / 60 (6.7%)		
40–65 years		80 / 143 (56.0%)	45 / 60 (75.0%)		
> 65 years		51 / 143 (35.6%)	11 / 60 (18.3%)		
Sex, n / N (%)	203			0.13	0.2
female		53 / 143 (37.0%)	15 / 60 (25.0%)		
male		90 / 143 (63.0%)	45 / 60 (75.0%)		
Ethnicity	203			0.3	0.4
Black		5 / 143 (3.5%)	0 / 60 (0%)		
Non-black		138 / 143 (96.5%)	60 / 60 (100%)		
Height (cm), Mean (SD)	203	171.1 (10.3)	171.0 (8.8)	0.8	0.9
Weight (kg), Mean (SD)	203	88.6 (22.1)	84.1 (17.8)	0.3	0.4
BMI (kg/m^2^), Mean (SD)	203	30.2 (6.9)	28.7 (5.7)	0.2	0.3
BMI Category, n / N (%)	203			0.7	0.8
< 20		3 / 143 (2.1%)	2 / 60 (3.3%)		
20–25		31 / 143 (22%)	14 / 60 (23.3%)		
25–30		49 / 143 (34%)	23 / 60 (38.3%)		
> 30		60 / 143 (42%)	21 / 60 (35.1%)		
SBP (mmHg), Mean (SD)	203	129.4 (18.6)	120.5 (19.8)	0.001	**0.003**
DBP (mmHg), Mean (SD)	203	76.9 (11.4)	70.1 (11.4)	< 0.001	** < 0.001**
BP Category, n / N (%)	203			0.006	**0.012**
Normal		35 / 143 (24.5%)	29 / 60 (48.0%)		
Elevated		22 / 143 (15.4%)	10 / 60 (17.0%)		
Stage I Hypertension		37 / 143 (25.9%)	9 / 60 (15.0%)		
Stage II Hypertension		49 / 143 (34.2%)	12 / 60 (20.0%)		
No BP Measurement		0 / 143 (0.0%)	0 / 60 (0.0%)		
CKD Stage, n / N (%)	203			0.022	**0.039**
G1		22 / 143 (15.0%)	12 / 60 (20.0%)		
G2		48 / 143 (34.0%)	13 / 60 (21.7%)		
G3a		36 / 143 (25.0%)	16 / 60 (26.7%)		
G3b		27 / 143 (19.0%)	6 / 60 (10.0%)		
G4		9 / 143 (6.3%)	12 / 60 (20.0%)		
G5		1 / 143 (0.7%)	1 / 60 (1.6%)		
Encephalopathy, n / N (%)	203			> 0.9	> 0.9
No		141 / 143 (98.6%)	59 / 60 (98.3%)		
Yes		2 / 143 (1.4%)	1 / 60 (1.7%)		
Ascites, n / N (%)	203			< 0.001	** < 0.001**
Not Present		143 / 143 (100.0%)	18 / 60 (30.0%)		
Present		0 / 143 (0%)	42 / 60 (70.0%)		
MELD Score, Mean (SD)	197	10.4 (3.3)	19.4 (8.6)	< 0.001	** < 0.001**
(Missing, n)		1	5		
Hepatic Dysfunction, n / N (%)	197			< 0.001	** < 0.001**
MELD ≤ 15		130 / 142 (91.5%)	18 / 55 (33.0%)		
MELD > 15		12 / 142 (8.5%)	37 / 55 (67.0%)		
(Missing, n)		1	5		
Child–Pugh Class, n / N (%)	203			< 0.001	** < 0.001**
Class A		143 / 143 (100.0%)	6 / 60 (10.0%)		
Class B		0 / 143 (0.0%)	45 / 60 (75.0%)		
Class C		0 / 143 (0.0%)	9 / 60 (15.0%)		
Serum Creatinine (mg/dL), Mean (SD)	203	1.36 (0.67)	1.14 (0.61)	< 0.001	**0.002**
Serum Cystatin C (mg/L), Mean (SD)	203	1.60 (0.65)	1.68 (0.78)	0.8	0.9
Serum albumin (g/dL), Mean (SD)	202	4.20 (0.41)	3.24 (0.58)	< 0.001	< 0.001
(Missing, n)		0	1		
mGFR,^f^ Mean (SD)	203	62.5 (26.5)	59.9 (31.5)	0.3	0.4
eGFRcr(ASR),^f^ Mean (SD)	203	61.6 (26.9)	77.3 (31.2)	< 0.001	**0.002**
eGFRcr(AS),^f^ Mean (SD)	203	64.3 (27.1)	80.1 (30.9)	< 0.001	**0.002**
eGFRcr-cys(ASR),^f^ Mean (SD)	203	54.5 (24.2)	61.5 (28.9)	0.10	0.2
eGFRcr-cys(AS),^f^ Mean (SD)	203	56.8 (25.0)	63.3 (29.6)	0.2	0.2
GFR_NMR_,^f^ Mean (SD)	203	60.3 (23.5)	59.2 (25.4)	0.7	0.8

*Abbreviations*: *AS* age and sex, *ASR* age, sex and race, *BMI* body mass index, *BP* blood pressure, *CKD* chronic kidney disease, *CP class* Child–Pugh class, *DBP* diastolic blood pressure, *eGFR* estimated glomerular filtration rate, *MELD* Model for End-stage Liver Disease, *mGFR* measured glomerular filtration rate, *SBP* systolic blood pressure, *SD* standard deviation

^a^Two (out of 205) samples had no assigned Child–Pugh (CP) score and were therefore omitted from the calculations (see Fig. 1); ^b^Preserved Liver Function, defined as CP class A without ascites; ^c^Reduced Liver Function, defined as CP class A with ascites, CP class B or CP class C;^d^Statistical tests performed: chi-square test of independence, Wilcoxon rank-sum test, Fisher’s exact test; ^e^Benjamini & Hochberg correction for multiple testing; ^f^ ml/min/1.73 m^2^ of body-surface area

### Statistical analysis

Data were integrated and prepared according to Stämmler et al. [17]. All calculations, performance evaluation and statistical tests were performed within R 4.0.2 [24]. Most metrics were calculated with *ModelMetrics* (Version 1.2.2.2) [25]. Data structures were handled with *data.table* (Version 1.13.2) [26] and *archivist* (Version 2.3.4) [27]. Bootstrap procedures were implemented via the *boot* package (Version 1.3–25) [28, 29]. Visualization was performed mainly with *ggplot2* (Version 3.3.2) [30]. Descriptive summary tables were created via the *gtsummary* (Version 1.3.5) package [31].

Key performance indicators (KPIs) for performance evaluation were selected as previously described [17]. Comparison of KPI performances by the different equations, overall and according to liver function, was performed with the following statistical tests. Comparison of IQR (precision) was performed by the boostrap method. Comparison of median bias was performed by the Wilcoxon-signed rank test [32, 33]. Comparison of accuracy measures (P30, P20, P15 and P10) was assessed by the McNemar’s chi square test [34]. All p-values were adjusted for multiple testing by Benjamini–Hochberg method (q-values). Statistical significance was determined by a p-value (adjusted) < 0.05.

The proportion of correct CKD staging (G1, G2, G3a, G3b, G4 and G5) by the different eGFR equations, compared to mGFR, was calculated in the whole cohort and within subgroups (defined according to hepatic or renal dysfunction). Pairwise comparison of CKD staging by GFR_NMR_ vs. that by other equations was performed using the McNemar’s test. In case of incorrect CKD staging, the proportion of underestimation of CKD severity (i.e., an overestimation of GFR leading to assign a better CKD stage than that assigned based on mGFR) or overestimation of CKD severity (i.e., an underestimation of GFR leading to assign a worse CKD stage than that assigned based on mGFR) was evaluated for the different eGFR equations.

## Results

### Patients’ characteristics

A total of 205 serum samples of patients with CLD were included in the performance analysis of eGFR equations. Performance evaluation was performed in the whole cohort and according to liver function (‘preserved’ versus ‘reduced’ liver function), based on CP scoring and the presence or absence of ascites (Fig. 1). Out of 205 samples, two could not be assigned a CP score and were excluded from the analysis per liver function. A total of 143/203 (70.4%) samples were assigned to the preserved liver function group and 60/203 (29.6%) to the reduced liver function group (Fig. 1). Sample distribution per center showed a higher proportion of patients with severe liver dysfunction among CLD samples from Lyon (35/50 [70.0%]) compared to Rochester (25/155 [16.1%]) (Fig. 1). This was confirmed by evaluating liver dysfunction severity according to the MELD score, CP scoring classification, and the ascites status of patients (Figure S1). This agrees with the observation that most patients from Lyon were candidates for OLT, while patients from Rochester were mostly outpatients (thus with expected lower CLD severity). Patients’ characteristics in the preserved and reduced liver function subgroups are described in Table 1. Mean GFR estimated by the various equations [eGFRcr(ASR), eGFRcr(AS), eGFRcr-cys(ASR), eGFRcr-cys(AS), GFR_NMR_] in both groups of CLD patients is also presented, along with mean mGFR (Table 1).

### Overall performance of eGFR equations

**Fig. 2:**
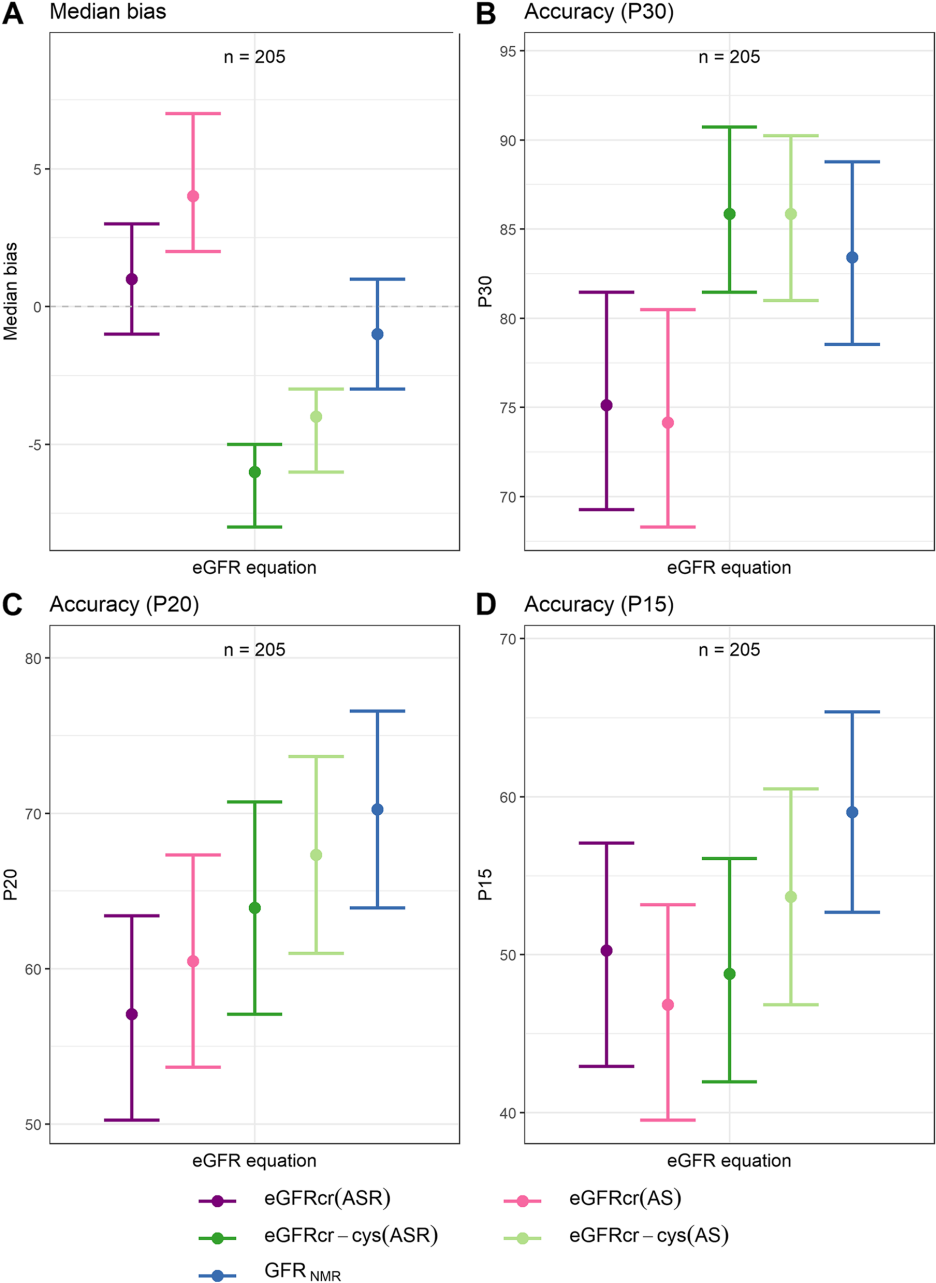
Key performance indicators (KPI) of eGFR equations in the whole dataset (*n* = 205). KPI evaluated were: median bias of eGFR to mGFR (**A**) and accuracy of eGFR measured as the percentage of samples with eGFR within 30% (P30) (**B**), 20% (P20) (**C**), and 15% (P15) (**D**) of mGFR. Error bars indicate bootstrapped 95% confidence interval (*n* = 1000). Dots represent the KPI of all data points. Each eGFR equation is represented by a different color, as indicated

Global performance of GFR_NMR_ was compared to that of the four CKD-EPI equations on *n* = 205 collected samples. Median bias of GFR_NMR_ was significantly different from that of all four CKD-EPI equations (Table 2; *p*-values between 0.0024 and < 0.0001). Median Bias was lowest for GFR_NMR_ and eGFRcr(ASR), with -1 (-3 to 1) and 1 (-1 to 3) ml/min/1.73 m^2^, respectively (Table 2 and Fig. 2A). Moreover, the race-free GFR_NMR_ equation demonstrated a lower median bias than its race-free CKD-EPI counterparts eGFRcr(AS) (4 ml/min/1.73 m^2^, *p* < 0.0001) and eGFRcr-cys(AS) (-4 ml/min/1.73 m^2^, *p* = 0.0024) (Table 2 and Fig. 2A).

**Table 2:** KPIs overall (*n* = 205) and by liver function (preserved liver function, *n* = 143; reduced liver function, *n* = 60). (To be placed after ‘Results’ subsection ‘Overall performance of eGFR equations’)

KPI	Equation	Overall	Preserved Liver Function (PLF)	Reduced Liver Function (RLF)	Difference in KPI between RLF and PLF
Precision: IQR^a^	eGFRcr(ASR)	19 [12–22]*	12.5 [10.5–16.5]	29.25 [23–35.5]*	-16.75 [-23.5–-8.01]
	eGFRcr(AS)	19 [12–22.97]*	13 [10.5–16]	31.5 [24–37.74]*	-18.5 [-25.25–-10.26]
	eGFRcr-cys(ASR)	14 [12–17]	**10 [9–12.5]**	15.25 [10.25–21.24]	-5.25 [-10.75–0.75]
	eGFRcr-cys(AS)	**13 [10–15]**	11 [9–14]	**13.5 [9–21]**	-2.5 [-10.25–2.5]
	GFR_NMR_	14 [12–16]	14 [11–16]	14.5 [10–22.49]	**-0.5 [-8.99–4]**
Bias: median bias^b^	eGFRcr(ASR)	1 [-1–3]*	-3 [-5–-1]	13 [9–25]*	-16 [-28–-11]
	eGFRcr(AS)	4 [2–7]*	1 [-2–2]*	16.5 [12–28.99]*	-15.5 [-28–-10.01]
	eGFRcr-cys(ASR)	-6 [-8–-5]*	-7 [-10–-6]*	2 [-2.5–4.5]*	-9 [-13–-4.5]
	eGFRcr-cys(AS)	-4 [-6–-3]*	-6 [-7–-4]*	2 [0–5]*	-8 [-11–-5]
	GFR_NMR_	**-1 [-3–1]**	-2 [-4–0]	**1 [-3.5–2.5]**	**-3 [-5–2]**
Accuracy: P10^c^	eGFRcr(ASR)	35.61 [29.27–41.95]	41.96 [33.57–49.65]	21.67 [11.67–31.67]	20.29 [6.63–32.55]
	eGFRcr(AS)	38.54 [31.22–44.88]	**46.15 [37.76–53.85]**	21.67 [11.67–31.67]	24.49 [10.73–37.28]
	eGFRcr-cys(ASR)	31.22 [24.88–37.07]	30.77 [23.08–38.46]	31.67 [20–43.33]	**-0.9 [-15.52–13.29]**
	eGFRcr-cys(AS)	35.12 [28.29–41.45]	35.66 [27.97–43.36]	33.33 [21.67–45]	2.33 [-12.02–16.09]
	GFR_NMR_	**41.95 [35.12–48.29]**	42.66 [34.27–51.05]	**41.67 [30–55]**	**0.99 [-13.74–15.35]**
Accuracy: P30^c^	eGFRcr(ASR)	75.12 [69.28–81.46]*	**88.81 [83.22–93.71]**	45 [33.33–56.67]*	43.81 [30.48–57.04]
	eGFRcr(AS)	74.15 [68.29–80.49]*	87.41 [82.52–92.31]	45 [33.33–56.67]*	42.41 [29.35–55.64]
	eGFRcr-cys(ASR)	**85.85 [81.46–90.72]**	**88.81 [83.22–93.71]**	**78.33 [66.67–88.33]**	**10.48 [-0.64–22.57]**
	eGFRcr-cys(AS)	**85.85 [80.99–90.24]**	**88.81 [83.22–93.71]**	**78.33 [66.67–88.33]**	**10.48 [-0.48–22.58]**
	GFR_NMR_	83.41 [78.54–88.78]	87.41 [82.52–92.99]	73.33 [61.67–85]	14.08 [1.71–26.61]

*Abbreviations*: *IQR* interquartile range, *KPI* key performance indicator, *PLF* preserved Liver Function; *RLF* Reduced Liver Function

^a^Precision defined as the interquartile range (IQR) of the difference to mGFR (ml/min/1.73 m^2^); ^b^Bias defined as the median difference to mGFR (ml/min/1.73 m^2^); ^c^P10 and P30 accuracy defined as the percentage of samples within an error tolerance to mGFR of 10% (P10) or 30% (P30) (%). Numbers in brackets show the bootstrapped 95% confidence intervals (*n* = 1000). Bold marked values show the best value for the given KPI over all five equations in the given subgroup. Symbol * indicates statistical significance (any *p*‐values < 0.05) in the pairwise tests against GFR_NMR_ for each KPI. The last column shows the difference between reduced and preserved liver function in the given KPI for each equation as a measure of disparity

Precision of GFR_NMR_ was significantly higher (i.e., lower IQR) than that of creatinine-based equations [eGFRcr(ASR) and eGFRcr(AS); *p* = 0.0025 and 0.0043] (Table 2) and was comparable to that of the creatinine- and cystatin C-based eGFR equations [eGFRcr-cys(ASR) and eGFRcr-cys(AS); *p* = 1 and 0.8495].

In addition, P30 and P20 accuracies of GFR_NMR_ were significantly higher than those of creatinine-only equations (*p*-values between 0.0142 and 0.0466) (Table 2, Table S1, Fig. 2B-C, and Figure S2) and were not different from those of creatinine and cystatin C CKD-EPI combined equations (*p*-values between 0.2031 and 0.5778). Although GFR_NMR_ P15 and P10 accuracies were the highest (59.02% for P15, 41.95% for P10) (Table 2, Table S1, Fig. 2D, and Figure S2), these differences did not reach significance (*p*-values between 0.0737 and 0.5387).

### Performance of eGFR equations by liver function

**Fig. 3:**
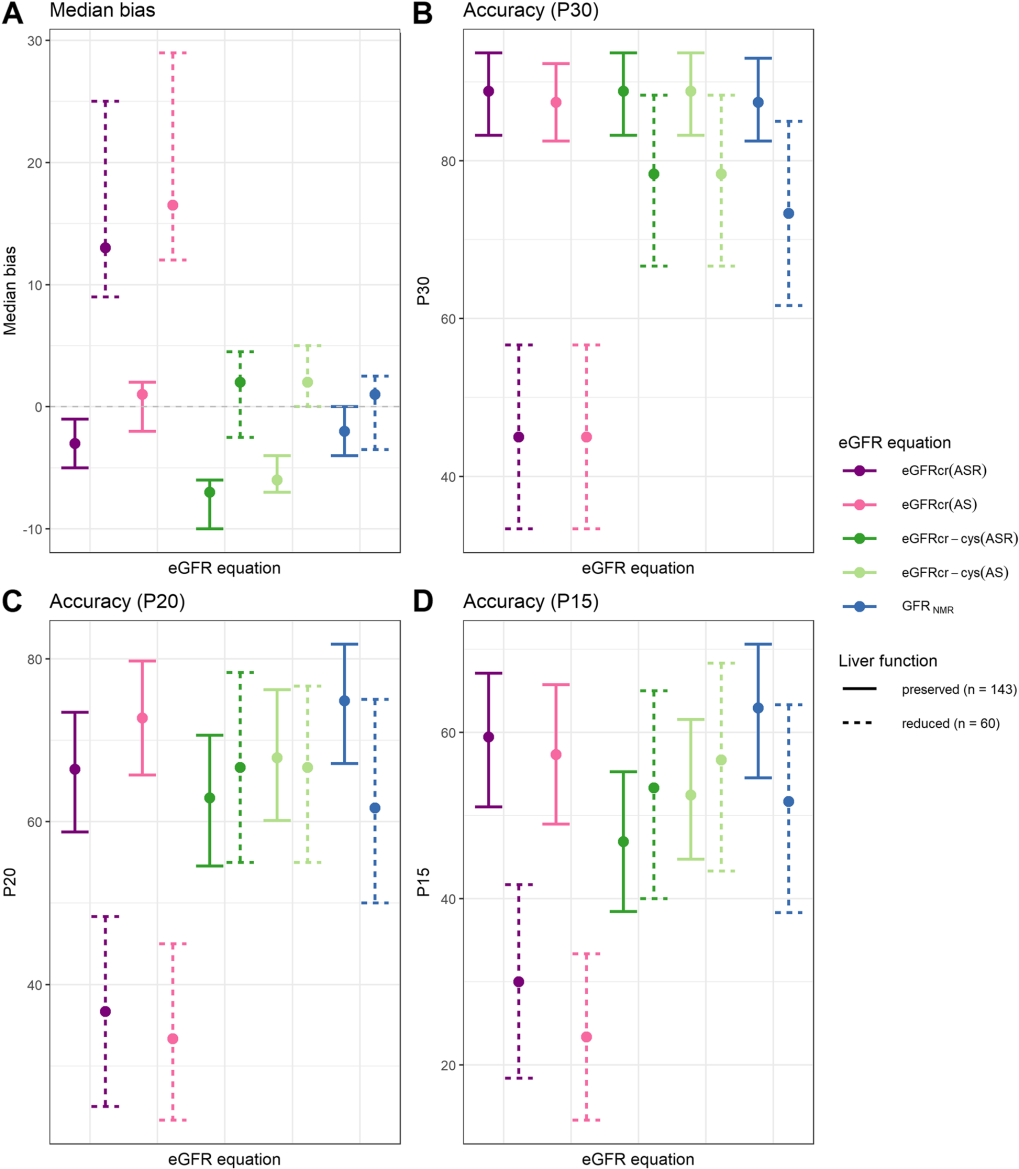
Key performance indicators (KPI) for each eGFR equation stratified by liver function (preserved vs. reduced). KPI evaluated were: median bias of eGFR to mGFR (**A**) and accuracy of eGFR measured as the percentage of samples with eGFR within 30% (P30) (**B**), 20% (P20) (**C**), and 15% (P15) (**D**) of mGFR. Error bars show the bootstrapped 95% confidence intervals (*n* = 1000). Solid lines of the error bars indicate the performance in the subgroup of patients with preserved liver function, while dashed lines indicate performance in patients with reduced liver function. Each eGFR equation is represented by a different color, whereas the tone of the color indicates the family of equations, with purple and pink encoding for creatinine-only equations [eGFRcr(ASR) and eGFRcr(AS)], dark and bright green encoding for creatinine and cystatin C-containing equations [eGFRcr-cys(ASR) and eGFRcr-cys(AS)], and blue encoding for the GFR_NMR_ equation

**Fig. 4:**
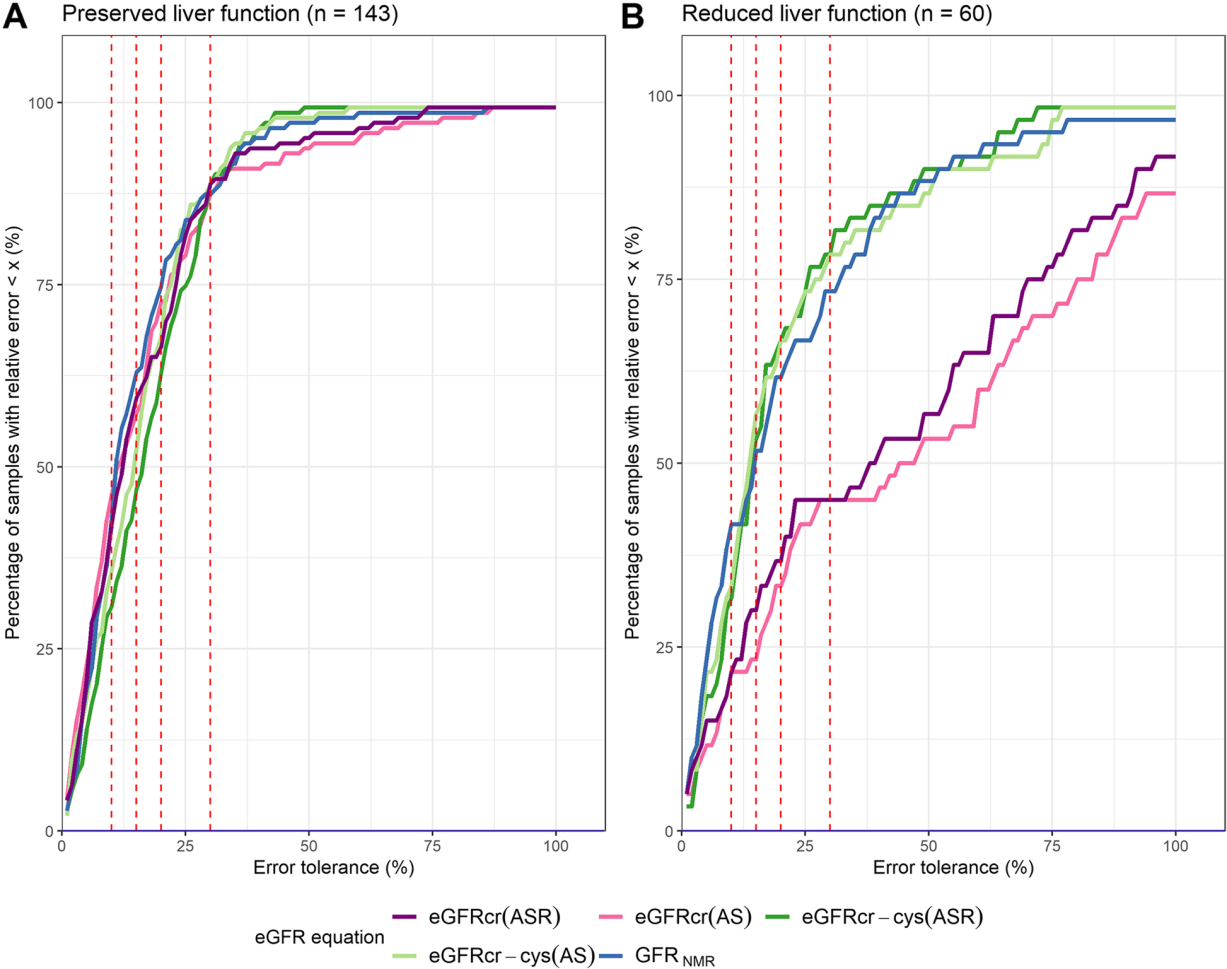
Accuracy levels of eGFR equations in patients with preserved and reduced liver function. Percentage of samples with eGFR within varying error tolerance compared to mGFR, for each eGFR equation among patients with preserved (**A**) and reduced (**B**) liver function. Each eGFR equation is represented by a different color. Red dashed vertical lines indicate error tolerance cutoffs at 10% (P10), 15% (P15), 20% (P20) and 30% (P30) (from left to right, respectively). Panel (**B**) demonstrates the inferior accuracy of creatinine-only eGFR equations in patients with reduced liver function

In CLD patients with preserved liver function (*n* = 143), all five eGFR equations revealed comparable performances as to precision, bias, and accuracy (Table 2, Table S1, Figs. 3 and 4A), with a few exceptions. Specifically, median bias of GFR_NMR_ (-2 ml/min/1.73 m^2^) was significantly different from that of eGFRcr(AS) (1 ml/min/1.73 m^2^, *p* = 0.0422), eGFRcr-cys(ASR) (-7 ml/min/1.73 m^2^, *p* < 0.0001) and eGFRcr-cys(AS) (-6 ml/min/1.73 m^2^, *p* < 0.0001) (Table 2 and Fig. 3A).

**Fig. 5:**
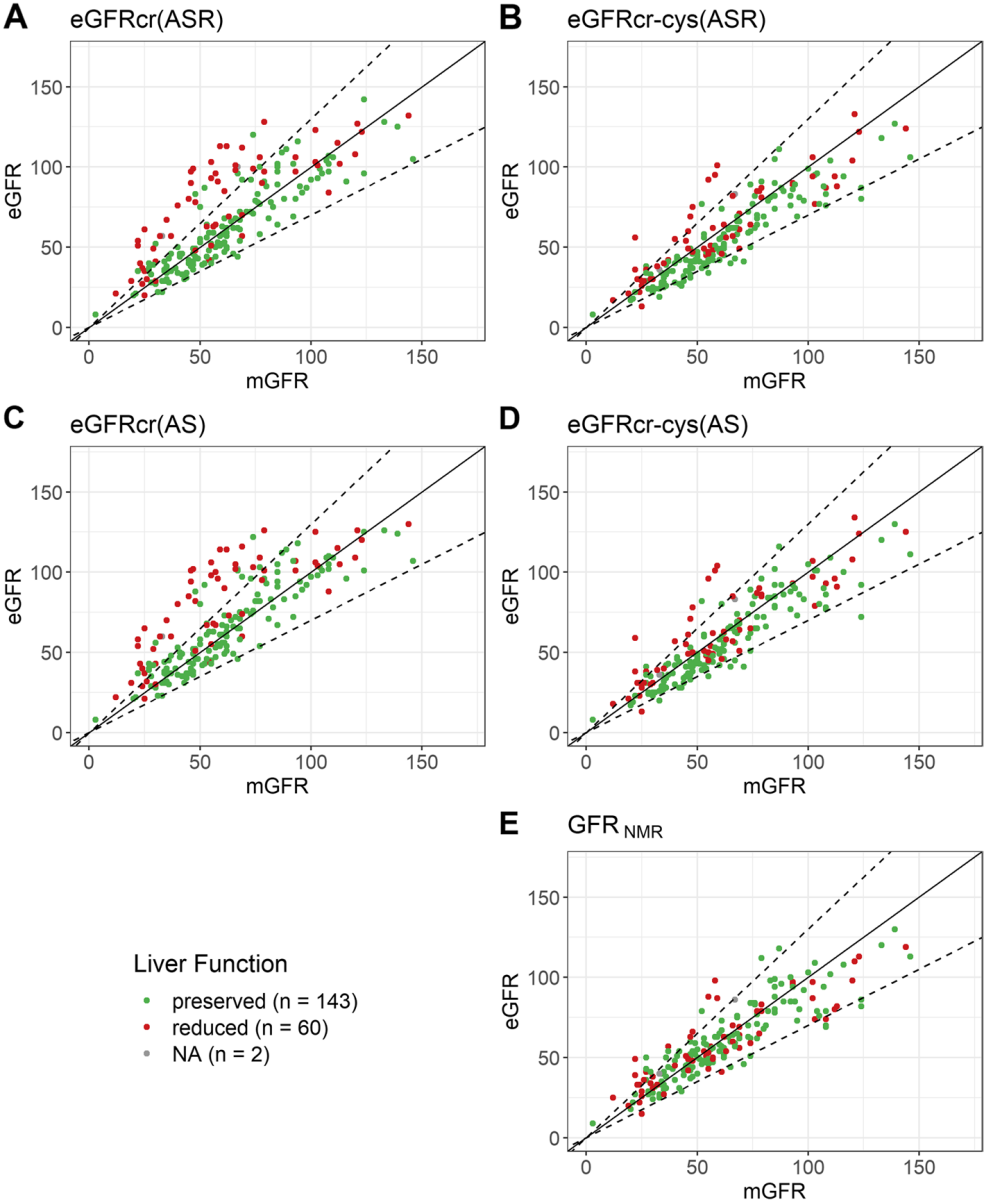
Scatterplot of estimated GFR (eGFR) versus measured GFR (mGFR) according to liver function. Estimated GFR calculated by each eGFR equation (**A-E**) is shown relative to the respective mGFR. The solid line indicates identity. Dashed black lines indicate P30 boundaries (values within identity and the dashed borders are considered within 30% of mGFR). Red dots indicate patients with reduced liver function (Child–Pugh Class A with Ascites, or Child–Pugh Class B, or Child–Pugh Class C; *n* = 60). Green dots indicate patients with preserved liver function (Child–Pugh Class A without Ascites; *n* = 143)

In CLD patients with reduced liver function (*n* = 60), however, differences in performances were observed between the compared eGFR equations. While GFR_NMR_ performed as well as the creatinine-cystatin C combined CKD-EPI equations regarding precision and accuracy (*p*-values between 0.4114 and 1), it performed statistically significantly better than the creatinine-only equations as to precision, bias, and accuracy (Table 2, Table S1, Figs. 3 and 4B). The superior accuracy of the GFR_NMR_ and eGFRcr-cys equations over that of eGFRcr equations was statistically significant for P30, P20 and P15 (*p*-values between 0.0002 and 0.0206) (Table 2, Table S1, Figs. 3B-D and 4B). Moreover, GFR_NMR_ performed best as to median bias (1 ml/min/1.73 m^2^) compared to eGFRcr(ASR) (13 ml/min/1.73 m^2^, *p* < 0.0001), eGFRcr(AS) (16.5 ml/min/1.73 m^2^, *p* < 0.0001), eGFRcr-cys(ASR) (2 ml/min/1.73 m^2^, *p* = 0.0448), and eGFRcr-cys(AS) (2 ml/min/1.73 m^2^, *p* = 0.0007) (Table 2 and Fig. 3A). The superiority of GFR_NMR_ and eGFRcr-cys over eGFRcr in patients with reduced liver function was even visually apparent when directly comparing eGFR to the respective mGFR by scatter plot analysis (Fig. 5). This analysis further illustrated that the creatinine-based equations systematically overestimated GFR in patients with reduced liver function (Fig. 5A,C versus B,D,E, red dots). These results were confirmed when comparing patients according to their MELD score (Figure S3) or their ascites status (Figure S4).

In addition, we evaluated the difference in performance between both liver function status as an indicator of performance stability. GFR_NMR_ showed the lowest difference in performance between preserved and reduced liver function as to precision (-0.5 ml/min/1.73 m^2^), median bias (-3 ml/min/1.73 m^2^), and P10 accuracy (0.99%) (Table 2, last column). In line with the lowest difference observed between median bias in both liver disease severity groups, GFR_NMR_ showed a marginal under- and overestimation of GFR in preserved and reduced liver function (-2 and 1 ml/min/1.73 m^2^, respectively) (Table 2 and Fig. 3A). The creatinine-based equations showed the highest difference in all cases (Table 2 and Table S1, last columns). eGFRcr-cys(ASR) and eGFRcr-cys(AS) showed the lowest difference in P30 accuracy (Table 2, last column), and eGFRcr-cys(AS) showed the lowest difference in P20 and P15 accuracy (Table S1, last column).

### CKD Staging performance by eGFR equations

Proper staging of renal dysfunction (defined according to the Kidney Disease Outcomes Quality Initiative (KDOQI) guidelines; [35]) is essential for accurate prognosis and patient management. With this in mind, performance of eGFR equations to assign a correct CKD stage (G1, G2, G3a, G3b, G4 or G5), compared to mGFR-based CKD staging as reference, was evaluated in the whole cohort and in subgroups defined according to hepatic or renal dysfunction (i.e., preserved or reduced liver function, MELD score ≤ or > 15, absence or presence of ascites, mGFR ≥ or < 60 ml/min/1.73 m^2^) (Table 3).

**Table 3:** eGFR equation performance in enabling accurate CKD staging (G1, G2, G3a, G3b, G4, G5). (To be placed after ‘Results’ subsection ‘CKD Staging performance by eGFR equations’)

**Subgroup**	**Samples (N)**	**Equation**	**Correct CKD Staging**^a^	**Error in CKD Staging**	
				**Underestimation of CKD severity**	**Overestimation of CKD severity**
Overall	205	eGFRcr(ASR)	98 (47.8%)	64 (31.2%)	43 (21.0%)
		eGFRcr(AS)	104 (50.7%)	74 (36.1%)	27 (13.2%)
		eGFRcr-cys(ASR)	104 (50.7%)	23 (11.3%)	78 (38.0%)
		eGFRcr-cys(AS)	109 (53.2%)	31 (15.1%)	65 (31.7%)
		GFR_NMR_	120 (58.5%)	37 (18.1%)	48 (23.4%)
Preserved liver function	143	eGFRcr(ASR)	78 (54.5%)	26 (18.2%)	39 (27.3%)
		eGFRcr(AS)	84 (58.7%)	34 (23.8%)	25 (17.5%)
		eGFRcr-cys(ASR)	67 (46.9%)	8 (5.5%)	68 (47.6%)
		eGFRcr-cys(AS)	71 (49.7%)	14 (9.7%)	58 (40.6%)
		GFR_NMR_	85 (59.4%)	22 (15.4%)	36 (25.2%)
Reduced liver function	60	eGFRcr(ASR)	20 (33.3%)*	36 (60.0%)	4 (6.7%)
		eGFRcr(AS)	20 (33.3%)*	38 (63.3%)	2 (3.4%)
		eGFRcr-cys(ASR)	35 (58.3%)	15 (25.0%)	10 (16.7%)
		eGFRcr-cys(AS)	36 (60.0%)	17 (28.3%)	7 (11.7%)
		GFR_NMR_	33 (55.0%)	15 (25.0%)	12 (20.0%)
MELD ≤ 15	148	eGFRcr(ASR)	78 (52.7%)	37 (25.0%)	33 (22.3%)
		eGFRcr(AS)	80 (54.1%)	46 (31.1%)	22 (14.8%)
		eGFRcr-cys(ASR)	73 (49.3%)	12 (8.1%)	63 (42.6%)
		eGFRcr-cys(AS)	76 (51.4%)	20 (13.5%)	52 (35.1%)
		GFR_NMR_	87 (58.8%)	26 (17.6%)	35 (23.6%)
MELD > 15	49	eGFRcr(ASR)	18 (36.7%)	21 (42.9%)	10 (20.4%)
		eGFRcr(AS)	22 (44.9%)	22 (44.9%)	5 (10.2%)
		eGFRcr-cys(ASR)	26 (53.1%)	9 (18.4%)	14 (28.5%)
		eGFRcr-cys(AS)	28 (57.1%)	9 (18.4%)	12 (24.5%)
		GFR_NMR_	28 (57.1%)	9 (18.4%)	12 (24.5%)
Ascites not present	163	eGFRcr(ASR)	86 (52.8%)	35 (21.5%)	42 (25.7%)
		eGFRcr(AS)	93 (57.1%)	43 (26.4%)	27 (16.5%)
		eGFRcr-cys(ASR)	80 (49.1%)	11 (6.7%)	72 (44.2%)
		eGFRcr-cys(AS)	84 (51.5%)	18 (11.1%)	61 (37.4%)
		GFR_NMR_	97 (59.5%)	26 (16.0%)	40 (24.5%)
Ascites present	42	eGFRcr(ASR)	12 (28.6%)*	29 (69.0%)	1 (2.4%)
		eGFRcr(AS)	11 (26.2%)*	31 (73.8%)	0 (0.0%)
		eGFRcr-cys(ASR)	24 (57.1%)	12 (28.6%)	6 (14.3%)
		eGFRcr-cys(AS)	25 (59.5%)	13 (31.0%)	4 (9.5%)
		GFR_NMR_	23 (54.8%)	11 (26.2%)	8 (19.0%)
mGFR ≥ 60^b^	96	eGFRcr(ASR)	56 (58.3%)	21 (21.9%)	19 (19.8%)
		eGFRcr(AS)	59 (61.5%)	24 (25.0%)	13 (13.5%)
		eGFRcr-cys(ASR)	53 (55.2%)	4 (4.2%)	39 (40.6%)
		eGFRcr-cys(AS)	61 (63.5%)	6 (6.2%)	29 (30.3%)
		GFR_NMR_	57 (59.4%)	6 (6.2%)	33 (34.4%)
mGFR < 60^b^	109	eGFRcr(ASR)	42 (38.5%)*	43 (39.4%)	24 (22.1%)
		eGFRcr(AS)	45 (41.3%)*	50 (45.9%)	14 (12.8%)
		eGFRcr-cys(ASR)	51 (46.8%)	19 (17.4%)	39 (35.8%)
		eGFRcr-cys(AS)	48 (44.0%)	25 (23.0%)	36 (33.0%)
		GFR_NMR_	63 (57.8%)	31 (28.4%)	15 (13.8%)

^a^Correct CKD staging marks the number of samples an equation correctly predicted CKD staging (against staging by mGFR), and symbol * indicates statistical significance (any *p*‐values < 0.05) in pairwise McNemar’s tests of GFR_NMR_ against each CKD-EPI equation; ^b^ml/min/1.73 m^2^. Numbers in parenthesis represent corresponding percentage of total patients within the given subgroup. Additionally, when CKD staging was not correctly predicted, under- and overestimation of CKD severity (corresponding to over- and underestimation of eGFR, respectively) was evaluated. Percentages sum up to 100% over all three columns

In the overall population, GFR_NMR_ showed the best CKD staging (120/205 [58.5%] correct CKD stages), as well as a balanced proportion of under- and overestimation of CKD severity in case of incorrect CKD staging (37/205 [18.1%] and 48/205 [23.4%], respectively) (Table 3). While the race-free CKD-EPI equations (“AS”) slightly improved CKD staging compared to their “ASR” counterparts, they were both biased toward either underestimating (eGFRcr) or overestimating (eGFRcr-cys) CKD severity (Table 3).

In subgroup analyses, the best CKD staging was achieved by GFR_NMR_ and eGFRcr-cys(AS) (Table 3). In contrast, creatinine-based equations were the least performant, especially in subgroups with severe liver dysfunction (Table 3). As for the overall population, GFR_NMR_ showed a balanced proportion of under- and overestimation of CKD severity in most subgroups, except for renal dysfunction. Here, GFR_NMR_ overestimated CKD severity in 33/96 (34.4%) patients with mGFR ≥ 60 ml/min/1.73 m^2^ compared to only 6/96 (6.2%) underestimates of CKD severity in these patients. In patients with reduced renal function (mGFR < 60 ml/min/1.73 m^2^), all equations tended to either overestimate (eGFRcr-cys) or underestimate (eGFRcr, GFR_NMR_ to a lesser extent) CKD severity. Noteworthily, the creatinine-based equations were the most biased equations toward underestimating CKD severity in patients with reduced liver function and in patients with ascites (Table 3).

## Discussion

In patients who suffer from CLD, renal impairment has significant implications, not only as a predictor of survival in the MELD score, yet also on drug dosing, interventions, and assessment for OLT. The very nature of CLD with distorted creatinine metabolism and ascites-associated hemodynamic instability renders creatinine-based GFR estimation unreliable to determine renal insufficiency [5, 10, 13, 36]. This study therefore undertook a performance comparison of GFR_NMR_ and the most widely used eGFR equations in a population of patients with CLD with mGFR as gold standard reference.

When considering the whole CLD population, GFR_NMR_ showed the least median bias. In contrast, eGFR equations incorporating both creatinine and cystatin C (eGFRcr-cys) underestimated GFR, while creatinine-only eGFR equations overestimated GFR. Accuracy of GFR_NMR_ was superior to that of eGFRcr equations and comparable to that of eGFRcr-cys equations. In patients with severe liver dysfunction, the superiority of GFR_NMR_ over eGFRcr was further confirmed, and GFR_NMR_ showed the smallest difference in bias between patients with reduced and preserved liver function, demonstrating its robustness regardless of liver disease severity. Determination of CKD stage in CLD is of major importance to determine if a combined hepatic and kidney transplantation should be performed. Clinical decision making based on a more accurate and less biased eGFR equation like GFR_NMR_, irrespective of the degree of liver impairment, is essential to reliably evaluate the renal functional reserve (RFR) and better predict patient recovery after OLT.

Our findings confirm previous works demonstrating that creatinine-based equations significantly overestimate mGFR [5, 13, 15, 16, 36], and validate the benefit of cystatin C as a biomarker of renal function in CLD [15, 16, 37]. Hemodynamic instability seen in ascites, in combination with an altered creatinine physiology and muscle mass catabolism seen in liver failure, is a likely driver of the unreliability of eGFRcr in our CLD cohort. The addition of cystatin C and further biomarkers in the NMR constellation (valine, myo-inositol) seem to improve the bias and accuracy of GFR estimations in decompensated liver failure. Similar results were obtained by De Souza et al. in subgroup analyses on comparing eGFR equations based on creatinine to equations based on cystatin C alone or in combination with creatinine, in patients with increasing ascites severity and those with a MELD score > 15 [15]. Furthermore, these results are in accordance with other studies suggesting that patients with ascites are more likely to have an overestimation of their GFR with creatinine-based eGFR [13], and that cystatin C correlates with GFR in end-stage liver failure, giving a diagnostic advantage in the detection of lower GFR in patients with liver failure [38]. These results support the view that the use of creatinine-based equations to determine GFR in CLD is limited and tends to worsen in correlation with the degree of severity of liver disease. Although the addition of cystatin C can improve the accuracy and bias of estimation, further addition of myo-inositol and valine in GFR_NMR_ appears to further improve the estimation of GFR.

To our knowledge, this is the first study to examine NMR-derived GFR estimations in CLD patients. The strengths of this study were the use of gold standard reference for GFR and its multicenter nature. Our study presents a few limitations. First, only 42/205 (20.5%) enrolled patients were documented as having ascites, which resulted in an imbalanced group of patients with reduced liver function (*n* = 60) compared to those with preserved liver function (*n* = 143). Second, there were some differences in ascites diagnostic criteria between both centers that needed retrospective CP scoring harmonization. Although this allowed a uniformized CP scoring, it might have biased clinical CP class assignments as our measure of liver dysfunction. However, our findings were confirmed by analyses according to MELD score or ascites status, limiting overall risk of bias. Third, despite the international nature of this study, only five patients were self-declared as black. As a result, this study group may not reflect the racial diversity seen in most liver centers in the USA and limit our findings to Caucasian patient populations. Fourth, our sample set comprised only *n* = 10 patients with GFR < 30 mL/min/1.73 m^2^. Thus, further studies are warranted in patients in CKD stages G4/5 to fully validate the clinical value GFR_NMR_ in patients with very low GFR.

## Conclusions

Creatinine-based equations are inaccurate in estimating GFR in patients with chronic liver disease. Despite the incorporation of cystatin C into the equation, errors are still seen especially with regards to accurate staging of CKD and in patients with more advanced liver disease. This study demonstrated that additional metabolites measured by NMR spectroscopy improve on shortfalls of creatinine- and cystatin C-based equations, particularly with regards to accuracy and bias. Since more than a decade, diagnostic NMR spectroscopy as such is readily available in nationally operating central clinical reference laboratories for low-density lipoprotein particle quantification in advanced cardiovascular risk assessment. Hence, GFR_NMR_ can also be available soon for assessment of renal functional reserve in patients with advanced chronic liver disease in conventional overnight services.

## Supplementary Information

**Additional file 1: Table S1. **Additional key performance indicators (KPI) for accuracy, overall (*n*=205) and by liver function (preserved liver function, *n*=143; reduced liver function, *n*=60) (complementary to Table 2). **Figure S1**. Sample distribution according to liver function and center (Lyon, Rochester). Liver dysfunction was defined according to different methods: (A) Preserved and reduced liver function, defined in this study based on Child-Pugh scoring and ascites status, (B) Child-Pugh scoring class (A, B and C), (C) MELD score ≤ and > 15, and (D) absence and presence of ascites. **Figure S2.** Accuracy levels of eGFR equations in the whole data set (*n*=205). Percentage of samples within varying error tolerance compared to mGFR, for each eGFR equation (represented by a different color). Red dashed vertical lines indicate error tolerance cutoffs at 10% (P10), 15% (P15), 20% (P20) and 30% (P30) (from left to right, respectively). **Figure S3.** Key performance indicators of eGFR equations according to hepatic dysfunction based on MELD score (≤ and > 15). Solid lines indicate performance for samples with MELD ≤15 and dashed lines indicate performance for samples with MELD score >15. Each eGFR equation is represented by a different color. **Figure S4.** Key performance indicators of eGFR equations according to the ascites status (present or absent) for each eGFR equation. Solid lines indicate performance for subgroup of patients without ascites, dashed lines show performance for subgroup of patients with ascites. Each eGFR equation is represented by a different color.

## Data Availability

The datasets used and/or analyzed during the current study are available from the corresponding author on reasonable request.

## Abbreviations

CI: Confidence interval
CKD: Chronic kidney disease
CKD-EPI: Chronic Kidney Disease Epidemiology Collaboration
CLD: Chronic liver disease
CP score/class: Child-Pugh score/class
eGFR: Estimated glomerular filtration rate
GFR: Glomerular filtration rate
HE: Hepatic encephalopathy
IQR: Interquartile range
KPI: Key performance indicator
mGFR: Measured glomerular filtration rate
OLT: Orthoptic liver transplantation
MELD: Model of End-stage Liver Disease
