# Supplementary material for: Impact of race-independent equations on estimating glomerular filtration rate for the assessment of kidney dysfunction in liver disease
Source: BMC Nephrol. 2023 Mar 31;24:83. doi: 10.1186/s12882-023-03136-y (PMC10064726; doi:10.1186/s12882-023-03136-y)
Supplement: Supplementary file 1 — Additional file 1: Table S1. Additional key performance indicators (KPI) for accuracy, overall (n=205) and by liver function (preserved liver function, n=143; reduced liver function, n=60) (complementary to Table 2). Figure S1. Sample distribution according to liver function and center (Lyon, Rochester). Liver dysfunction was defined according to different methods: (A) Preserved and reduced liver function, defined in this study based on Child-Pugh scoring and ascites status, (B) Child-Pugh scoring class (A, B and C), (C) MELD score ≤ and > 15, and (D) absence and presence of ascites. Figure S2. Accuracy levels of eGFR equations in the whole data set (n=205). Percentage of samples within varying error tolerance compared to mGFR, for each eGFR equation (represented by a different color). Red dashed vertical lines indicate error tolerance cutoffs at 10% (P10), 15% (P15), 20% (P20) and 30% (P30) (from left to right, respectively). Figure S3. Key performance indicators of eGFR equations according to hepatic dysfunction based on MELD score (≤ and > 15). Solid lines indicate performance for samples with MELD ≤15 and dashed lines indicate performance for samples with MELD score >15. Each eGFR equation is represented by a different color. Figure S4. Key performance indicators of eGFR equations according to the ascites status (present or absent) for each eGFR equation. Solid lines indicate performance for subgroup of patients without ascites, dashed lines show performance for subgroup of patients with ascites. Each eGFR equation is represented by a different color. [file 12882_2023_3136_MOESM1_ESM.pdf]

SUPPLEMENTARY MATERIAL

TABLE S1. Additional key performance indicators (KPI) for accuracy, overall (n=205) and by liver function (preserved liver function, n=143; reduced liver function, n=60) (complementary to Table 2).

KPI	Equation	Overall	Preserved Liver Function (PLF)	Reduced Liver Function (RLF)	Difference in KPI between RLF and PLF
Accuracy: P15 [1]	eGFRcr(ASR)	50.24 [42.93–57.07]	59.44 [51.05–67.13]	30 [18.38–41.67]*	29.44 [16.21–43.1]
	eGFRcr(AS)	46.83 [39.51–53.17]	57.34 [48.97–65.73]	23.33 [13.33–33.33]*	34.01 [20.35–46.81]
	eGFRcr-cys(ASR)	48.78 [41.95–56.09]	46.85 [38.46–55.24]	53.33 [40–65]	-6.48 [-21.75–9.05]
	eGFRcr-cys(AS)	53.66 [46.83–60.49]	52.45 [44.76–61.54]	**56.67 [43.33–68.33]**	**-4.22 [-18.95–11.27]**
	GFR_{NMR}	**59.02 [52.68–65.37]**	**62.94 [54.55–70.63]**	51.67 [38.33–63.33]	11.27 [-2.66–26]
Accuracy: P20 [1]	eGFRcr(ASR)	57.07 [50.24–63.41]*	66.43 [58.74–73.43]	36.67 [25–48.33]*	29.77 [15.41–43.8]
	eGFRcr(AS)	60.49 [53.66–67.32]*	72.73 [65.73–79.72]	33.33 [23.33–45]*	39.39 [25.31–52.19]
	eGFRcr-cys(ASR)	63.9 [57.07–70.73]	62.94 [54.55–70.63]	**66.67 [55–78.33]**	-3.73 [-17.65–11.69]
	eGFRcr-cys(AS)	67.32 [60.98–73.66]	67.83 [60.14–76.22]	**66.67 [55–76.67]**	**1.17 [-12.22–15.52]**
	GFR_{NMR}	**70.24 [63.9–76.59]**	**74.83 [67.13–81.82]**	61.67 [50–75]	13.16 [-1.19–28.05]

[1] P15 and P20 accuracy is defined as the percentage of samples within an error tolerance to mGFR of 15% (P15) or 20% (P20) (%). Numbers in brackets show the bootstrapped 95% confidence intervals (n = 1000). Bold marked values show the best value for the given KPI over all five equations in the given subgroup. Symbol * indicates statistical significance (any *p*-values < 0.05) in the pairwise tests against GFR_{NMR} for each KPI. Relevant exact *p*-values are described in the text. The last column shows the difference between reduced and preserved liver function in the given KPI for each equation as a measure of disparity. Abbreviations: KPI, key performance indicator; PLF, preserved Liver Function; RLF, Reduced Liver Function.

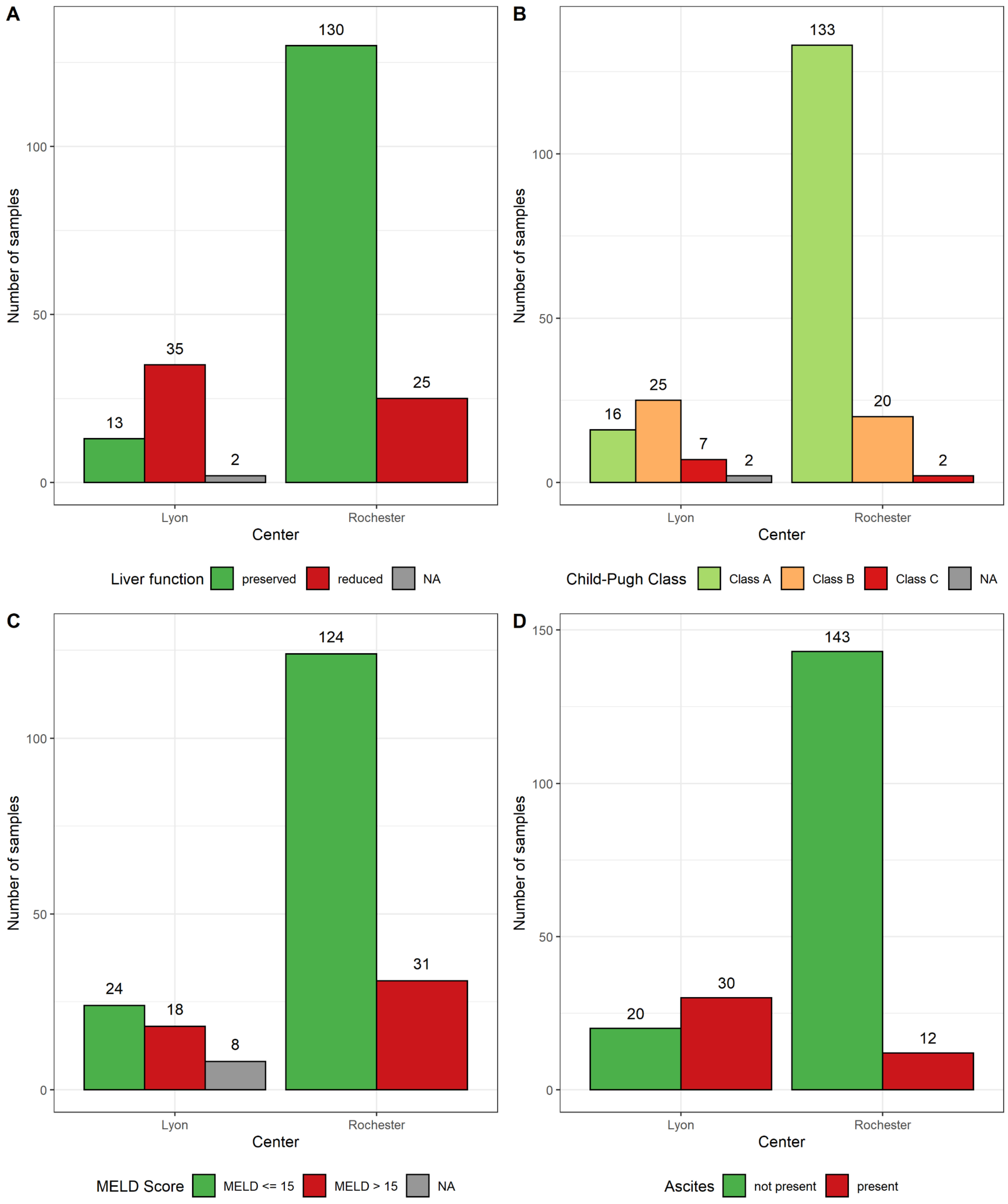

FIGURE S1. Sample distribution according to liver function and center (Lyon, Rochester). Liver dysfunction was defined according to different methods: (**A**) Preserved and reduced liver function, defined in this study based on Child-Pugh scoring and ascites status, (**B**) Child-Pugh scoring class (A, B and C), (**C**) MELD score ≤ and > 15, and (**D**) absence and presence of ascites.

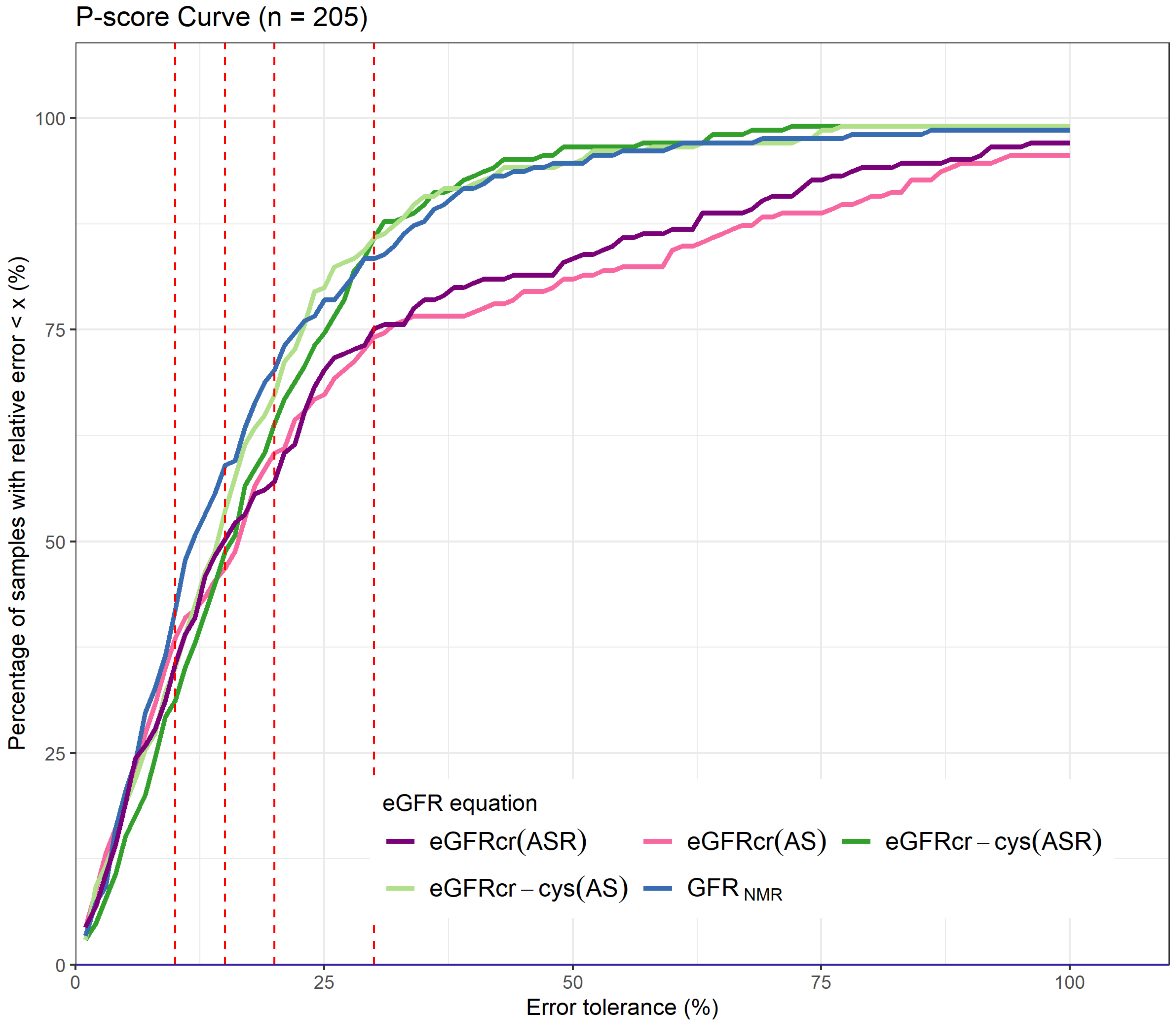

FIGURE S2. Accuracy levels of eGFR equations in the whole dataset (n=205). Percentage of samples within varying error tolerance compared to mGFR, for each eGFR equation (represented by a different color). Red dashed vertical lines indicate error tolerance cutoffs at 10% (P10), 15% (P15), 20% (P20) and 30% (P30) (from left to right, respectively).

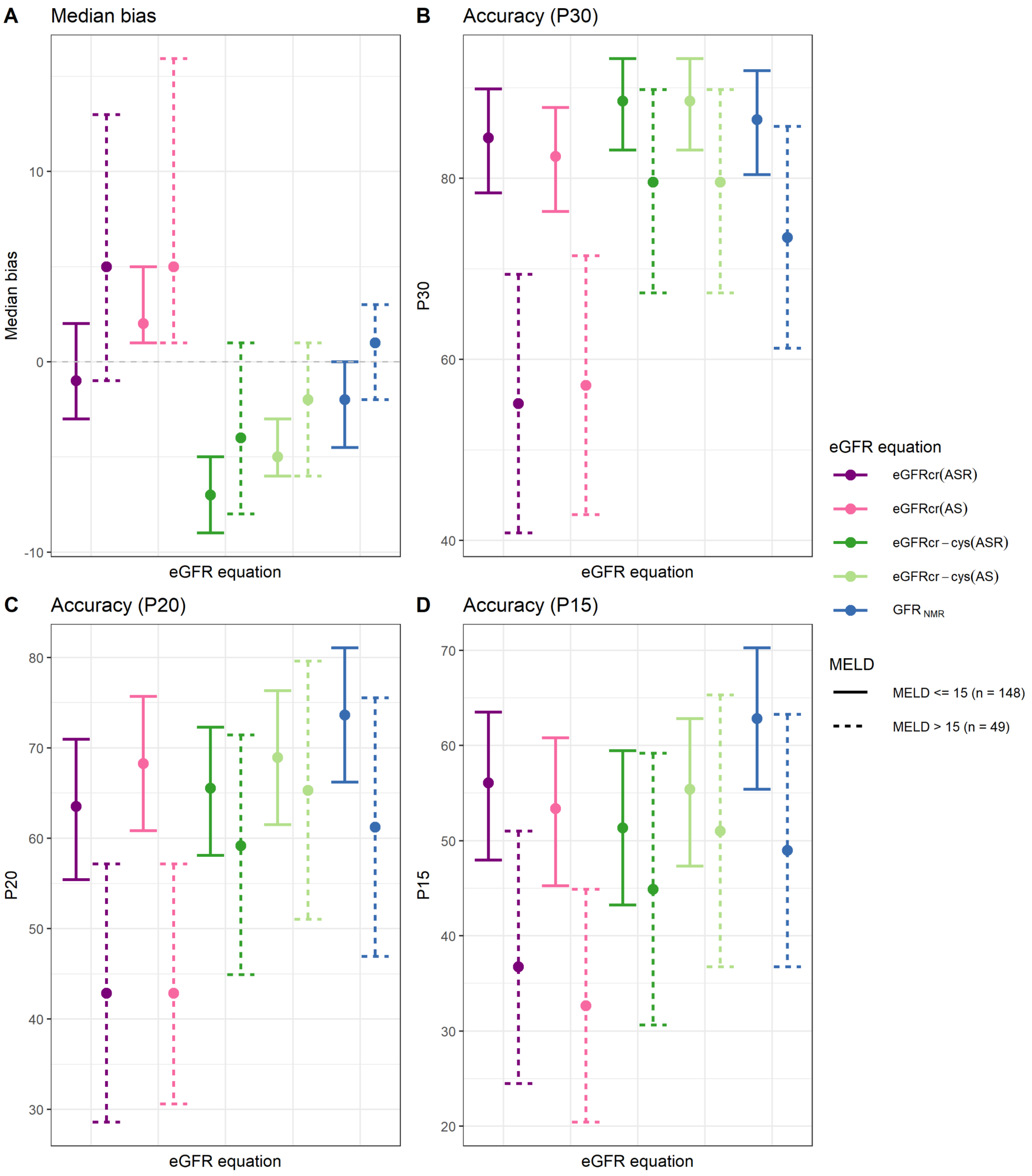

FIGURE S3. Key performance indicators of eGFR equations according to hepatic dysfunction based on MELD score (≤ and > 15). Solid lines indicate performance for samples with MELD ≤ 15 and dashed lines indicate performance for samples with MELD score > 15. Each eGFR equation is represented by a different color.

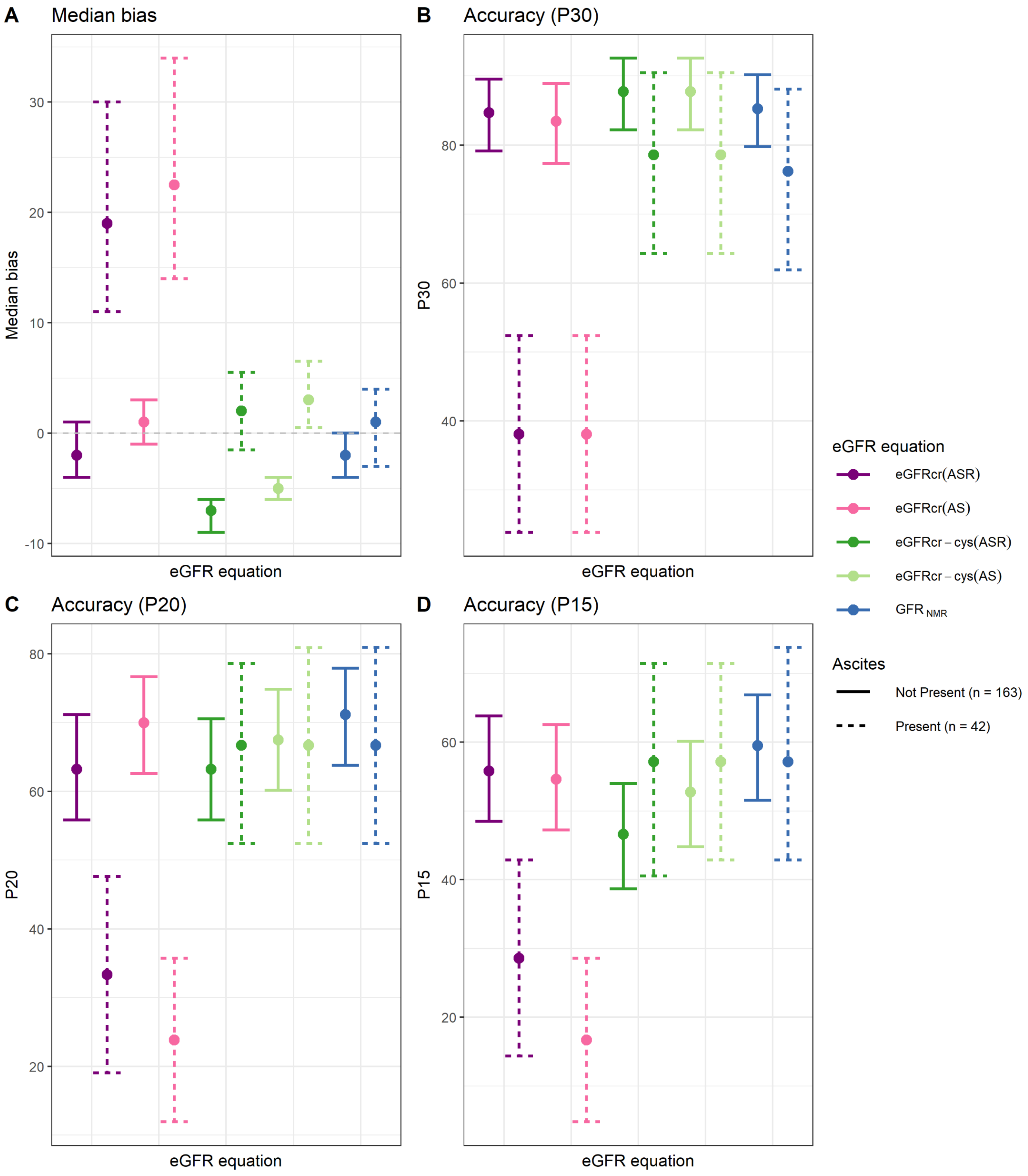

FIGURE S4. Key performance indicators of eGFR equations according to the ascites status (present or absent) for each eGFR equation. Solid lines indicate performance for subgroup of patients without ascites, dashed lines show performance for subgroup of patients with ascites. Each eGFR equation is represented by a different color.
